# Supplementary material for: A HepG2 Cell-Based Biosensor That Uses Stainless Steel Electrodes for Hepatotoxin Detection
Source: Biosensors (Basel). 2022 Mar 4;12(3):160. doi: 10.3390/bios12030160 (PMC8946082; doi:10.3390/bios12030160)
Supplement: Supplementary file 1 [file biosensors-12-00160-s001.zip › biosensors-1614531-supplementary.pdf]

Supporting information - A liver cell-based biosensor that uses stainless steel electrodes as substrate

Martin Rozman, Zala Štukovnik, Ajda Sušnik, Amirhossein Paksheresht, Matej Hočevar, Damjana Drobne and Urban Bren*

*Corresponding Author

Resazurin assay

The metabolic activity of HepG2 cells exposed to AFB1 was determined by the resazurin assay using weakly fluorescent resorufin. As a response to cellular metabolism, weak fluorescent resorufin oxidize to highly fluorescent resorufin.

The resazurin assay shows that AFB1 significantly reduces the metabolic activity of HepG2 cells at a concentration in excess of 20 μM. The reduction in metabolic activity can be explained by a decrease in the number of cells, where some of the cells undergo apoptosis due to cell damage.

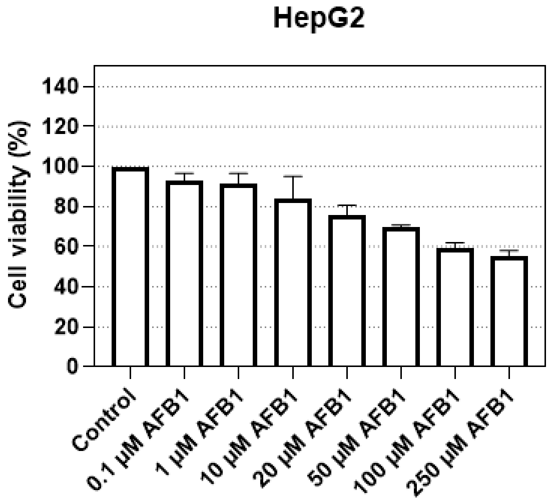

Figure S1: (Resazurin assay) The HepG2 cells viability after exposure to various concentrations of AFB1 (0, 0.1, 1, 10, 20, 50, 100 and 250 μM). The viability of the cells was determined by the Coomassie resazurin dye test. The results are given as mean values of the three measurements ± STD (*** $p<0.001$).

Fluorescence microscopy:

The following microphotographs were obtained in order to obtain cell viability before and after exposure to AFB1 and INH. These microphotograph were used for cell counting and are presented as Figure 3 in the main text.

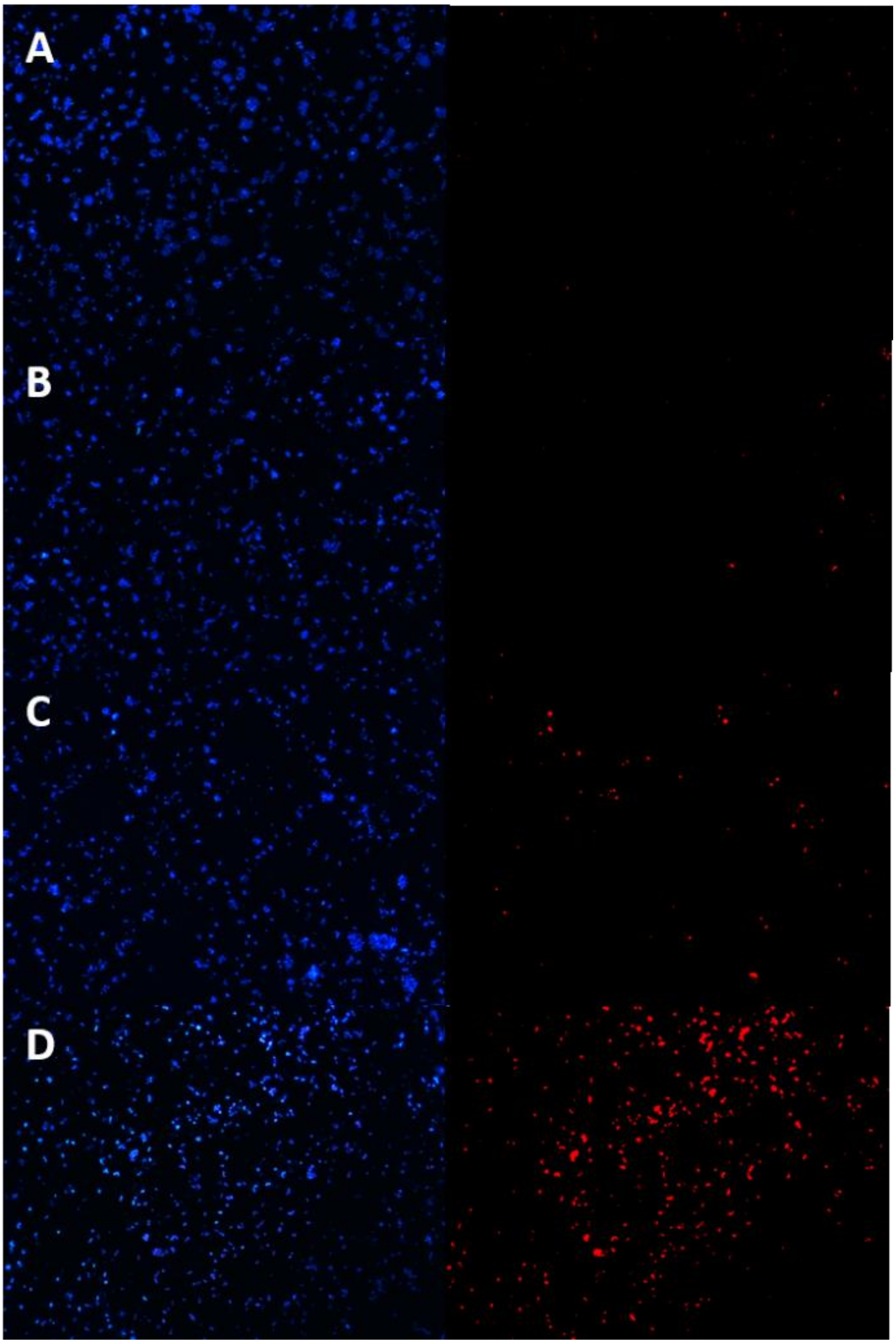

Figure S2: (Fluorescence microscopy) Photographs of fluorescent microscopy under the influence of AFB1 on the survival of HepG2 cells. A represents HepG2 cells exposed to 0 μM (control), B represents HepG2 cells exposed to 5 μM, C represents HepG2 cells exposed to 50 μM, and D represents the HepG2 cells exposed to 250 μM.

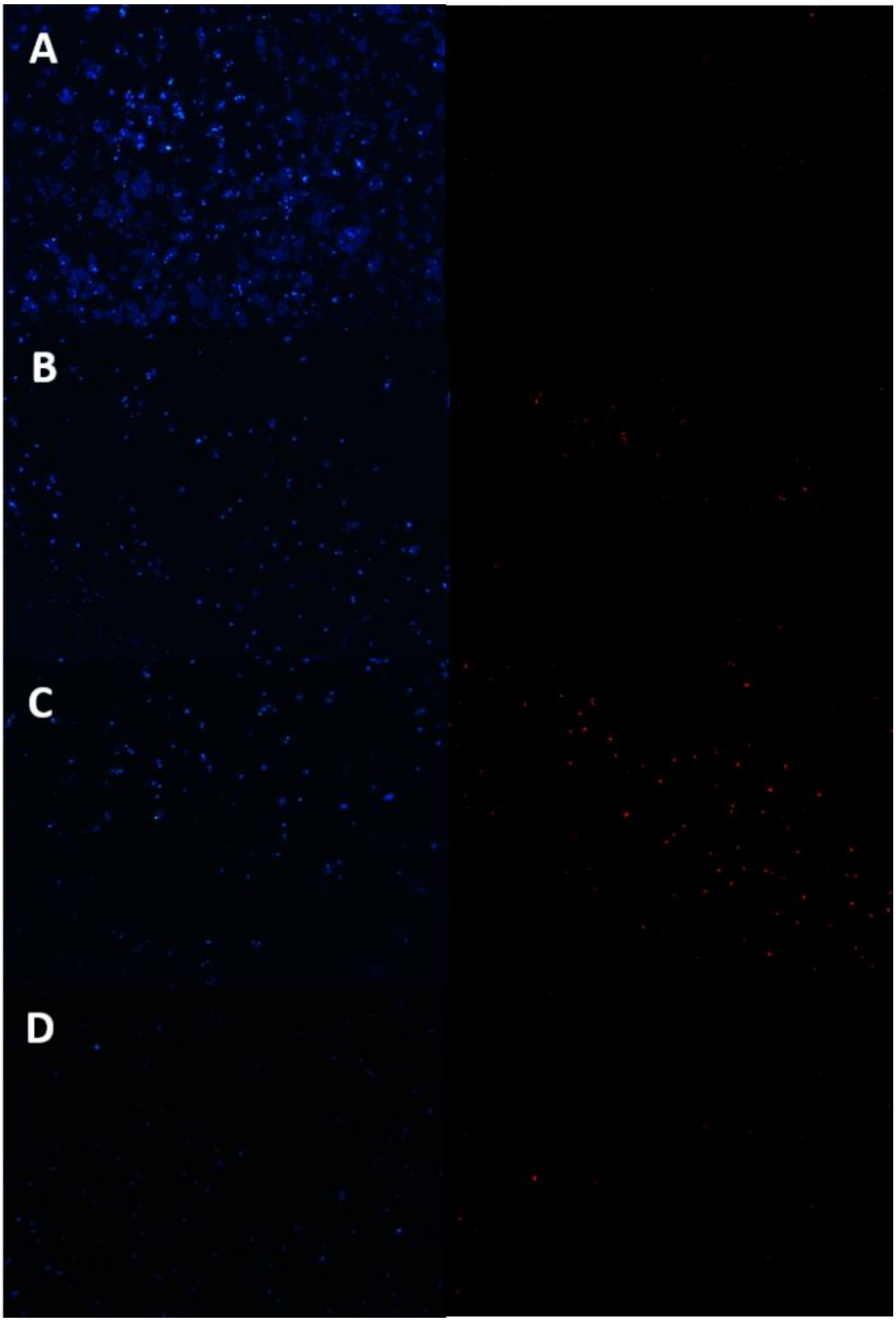

Figure S3: (Fluorescence microscopy) Photographs of fluorescent microscopy under the influence of INH on the survival of HepG2 cells. A represents HepG2 cells exposed to 0 mM (control), B represents HepG2 cells exposed to 5 mM, C represents HepG2 cells exposed to 50 mM, and D represents the HepG2 cells exposed to 250 mM.

Scanning electron microscopy:

The following electron microscopy microphotographs were taken during the morphological analysis of the suitability of the stainless steel surface as an electrode for the biosensor. For each figure; magnification, acceleration voltage and scale are given.

Aflatoxin B1:

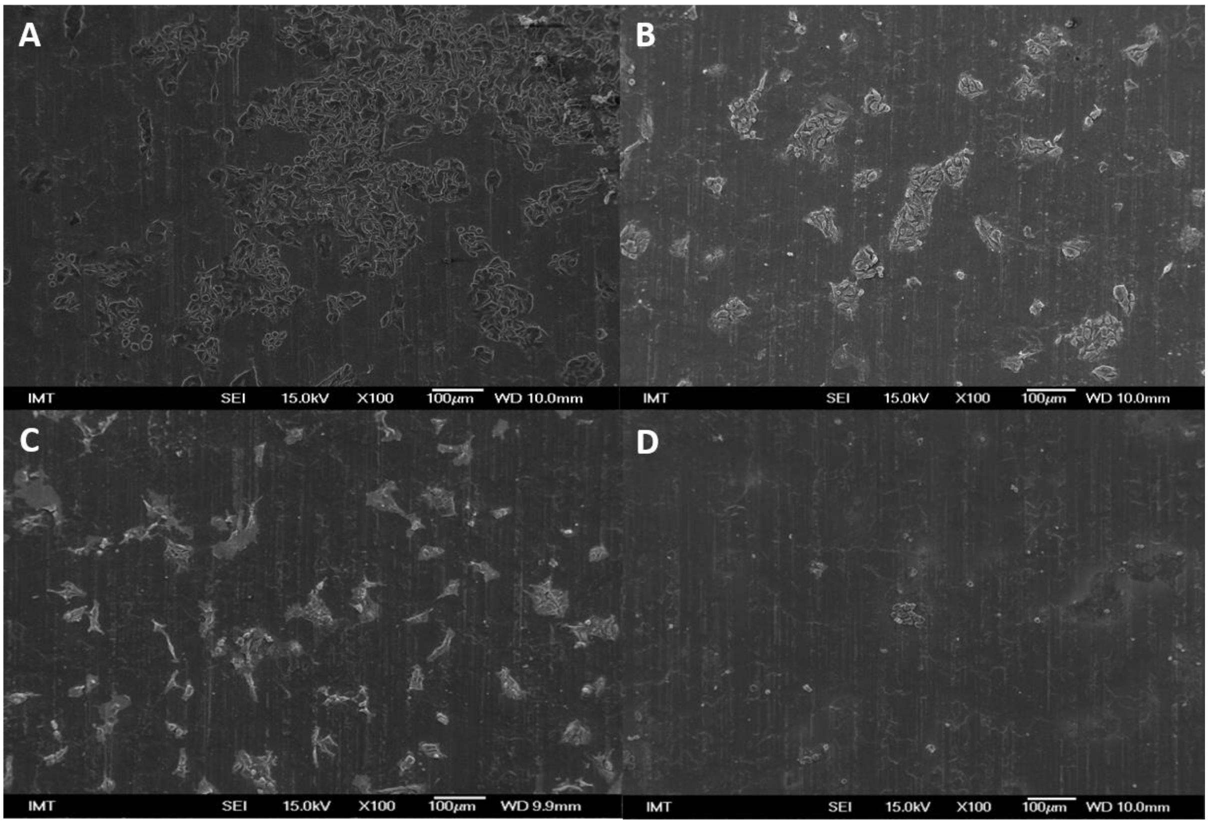

Figure S4: SEM photographs of the HepG2 cells exposed to increasing concentrations of the AFB1 at 100× magnification. A represents HepG2 cells exposed to 0 μm (control), B represents HepG2 cells exposed to 5 μm, C represents HepG2 cells exposed to 50 μm, and D represents the HepG2 cells exposed to 250 μm.

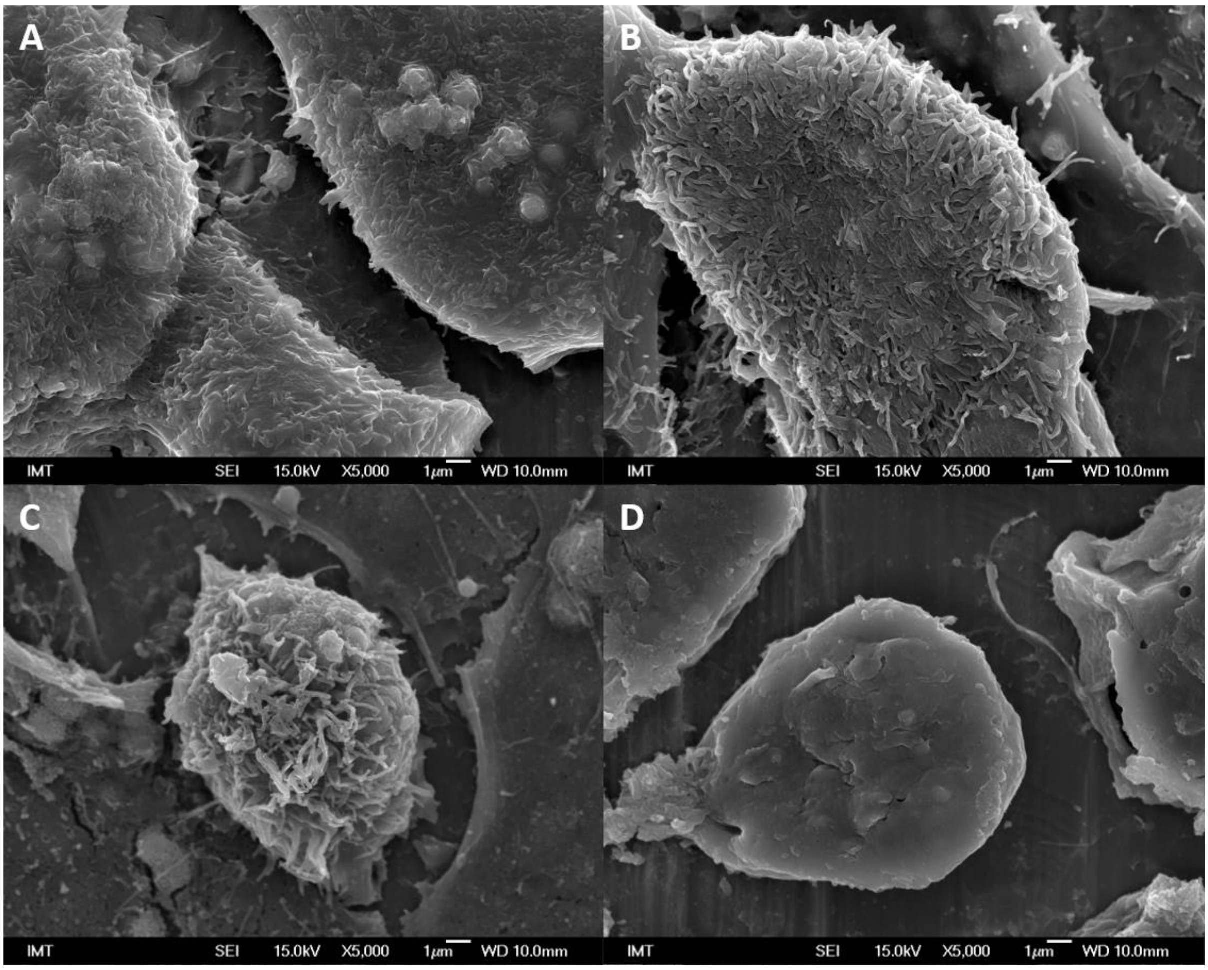

Figure S5: SEM photographs of the HepG2 cells exposed to increasing concentrations of the AFB1 at 5000× magnification. A represents HepG2 cells exposed to 0 μm (control), B represents HepG2 cells exposed to 5 μm, C represents HepG2 cells exposed to 50 μm, and D represents the HepG2 cells exposed to 250 μm.

Isoniazide:

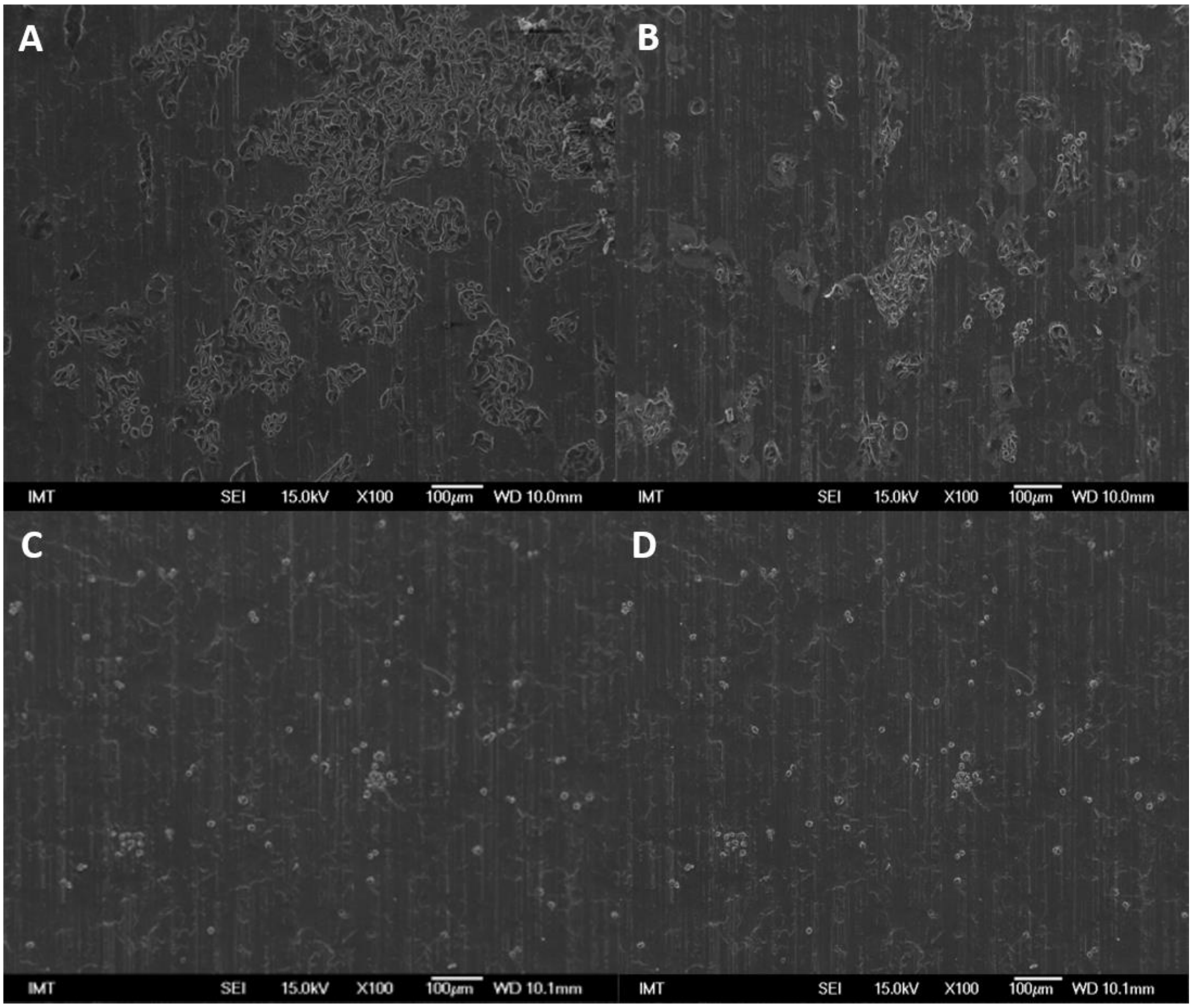

Figure S6: Photographs of scanning electron microscopy under the influence of INH on the morphology of human carcinoma hepG2 cells at 100× magnification. A represents HepG2 cells exposed to 0 μm (control), B represents HepG2 cells exposed to 5 μm, C represents HepG2 cells exposed to 50 μm, and D represents the HepG2 cells exposed to 250 μm.

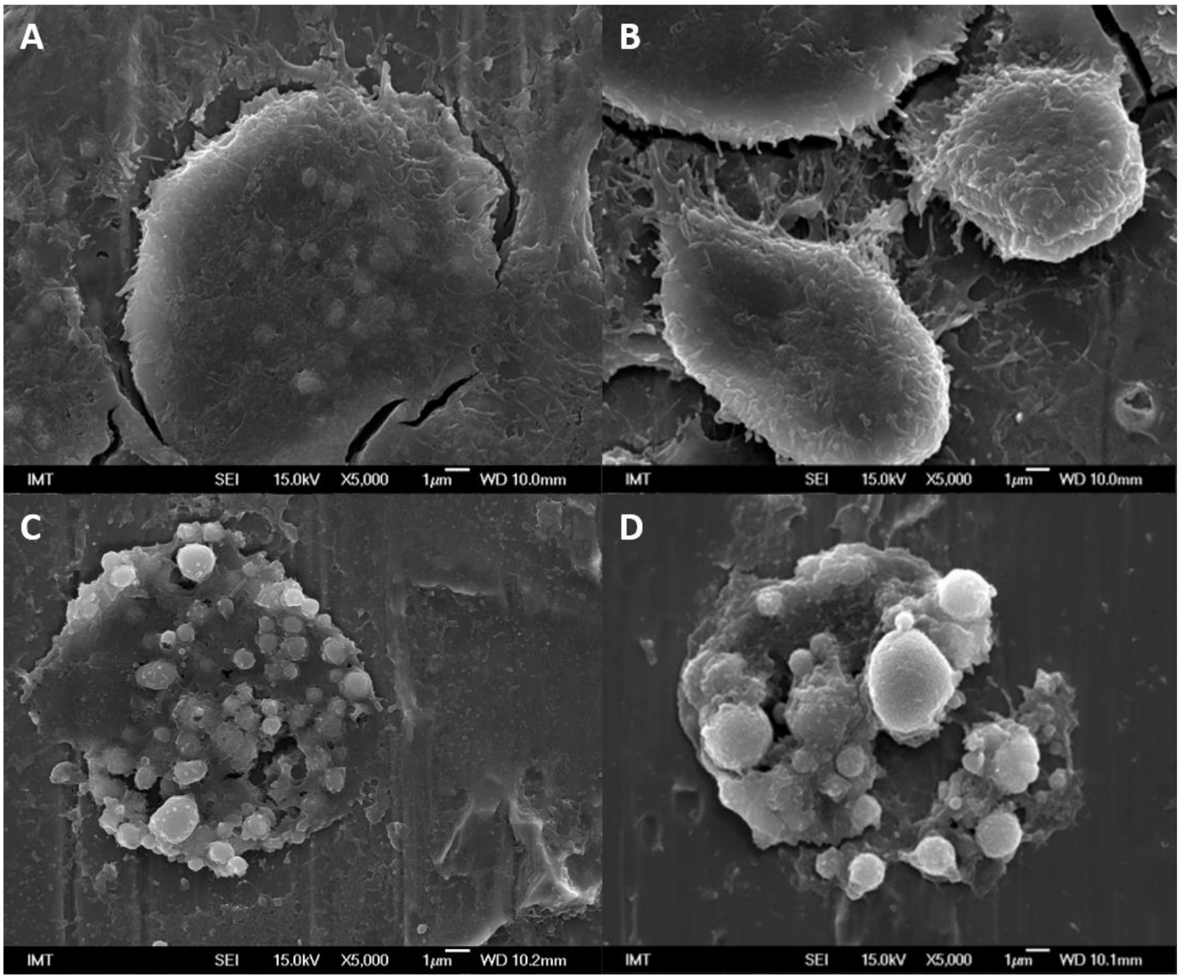

Figure S7: Photographs of scanning electron microscopy under the influence of INH on the morphology of human carcinoma hepG2 cells at 5000 × magnification A represents HepG2 cells exposed to 0 μm (control), B represents HepG2 cells exposed to 5 μm, C represents HepG2 cells exposed to 50 μm, and D represents the HepG2 cells exposed to 250 μm.

EIS circuit fitting

Presented here is the data from circuit fitting along with the appropriate Nyquist diagrams and circuits (Figure S8 and S9). The circuit data is available in the following tables (Figure S10 and S11).

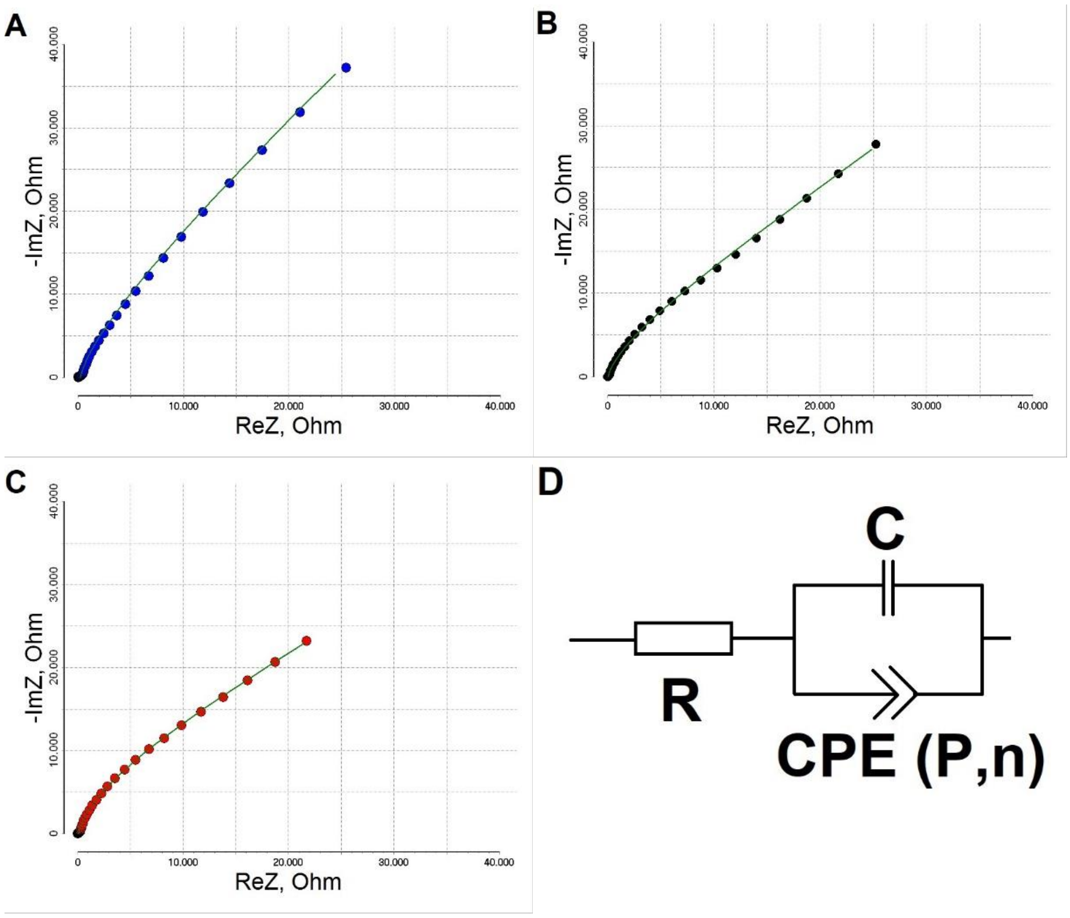

Figure S8: Fitted data using the circuit fitting presented in Figure 6 for actual EIS data obtained during the measurement of the assembled biosensor with negative control electrode (Figure S8-a), added aflatoxin B1 (AFB1) (Figure S8-b) and the positive control without cells on surface (Figure S8-c). In addition to the Nyquist plots, a circuit is presented as well (Figure S8-d) The color schematics are the same used as in the Figure 6a.

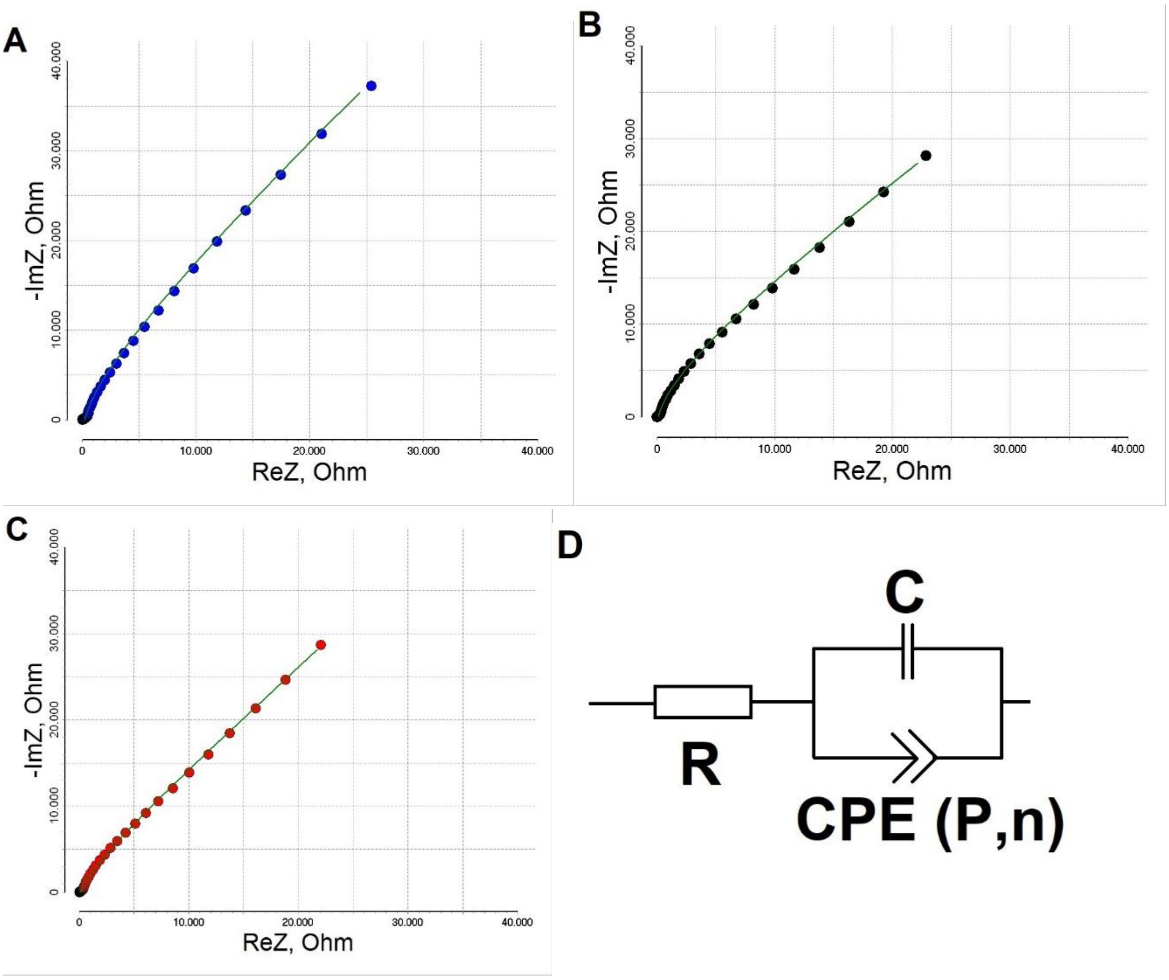

Figure S9: Fitted data using the circuit fitting presented in Figure 6 for actual EIS data obtained during the measurement of the assembled biosensor with negative control electrode (Figure S9-a), added Isoniazid (INH) (Figure S9-b) and the positive control without cells on surface (Figure S9-c). In addition to the Nyquist plots, a circuit is presented as well (Figure S8-d) The color schematics are the same used as in the Figure 6b.

Table S1: Fitting data for Aflatoxin B1:

parameter	negative	AFB1	positive
R [Ω]	268.93	79.186	124.7
C [F]	7.7695E-06	5.5211E-06	9.6087E-06
P [Ω^{-1}]	2.4361E-05	3.0769E-05	3.3803E-05
N parameter	0.5377	0.46859	0.42744

Table S2: Fitting data for Isoniazid:

parameter	negative	AFB1	positive
R [Ω]	264.82	136.82	159.86
C [F]	7.7642E-06	8.1713E-06	5.029E-06
P [Ω^{-1}]	2.4352E-05	3.095E-05	3.308E-05
